# Climatic Factors and the Incidence of Pyelonephritis During Pregnancy

**DOI:** 10.1155/S1064744995000688

**Published:** 1995

**Authors:** John D. Busowski, Ronald A. Chez

**Affiliations:** 1 Department of Obstetrics and Gynecology University of South Florida College of Medicine Tampa FL USA; 2 Department of Obstetrics and Gynecology University of South Alabama 2451 Fillingim Street Mobile AL 36617 USA

## Abstract

*Objective:* Numerous published reports have linked various disease states and pregnancy-related conditions with meteorologic factors such as weather, humidity, and temperature. The purpose of this study was to determine if temperature and dew point affect the incidence of pyelonephritis during pregnancy.

*Methods:* A retrospective chart review of a 4-year period from 1989 to 1992 was performed. The records of women who were diagnosed with pyelonephritis during pregnancy were abstracted for the dates of admission. The climatic records of the Tampa Bay area of Florida were obtained from the National Weather Service.

*Results:* The average, minimum, or maximum daily temperature or average daily dew point during the month of admission had no significant effect on the rate of pyelonephritis during pregnancy in the Tampa Bay area.

*Conclusions:* The rate of pyelonephritis during pregnancy per number of deliveries in the Tampa Bay area was not affected by the average, minimum, or maximum daily temperature or average daily dew point.


Infectious Diseases in Obstetrics and Gynecology 3:226–228 (1995)
© 1996 Wiley-Liss, Inc.

### Climatic Factors and the Incidence of Pyelonephritis During Pregnancy

**John D. Busowski and Ronald A. Chez**

*Department of Obstetrics and Gynecology, University of South Florida College of Medicine, Tampa, FL*

ABSTRACT

***Objective:*** Numerous published reports have linked various disease states and pregnancy-related conditions with meteorologic factors such as weather, humidity, and temperature. The purpose of this study was to determine if temperature and dew point affect the incidence of pyelonephritis during pregnancy.

***Methods:*** A retrospective chart review of a 4-year period from 1989 to 1992 was performed. The records of women who were diagnosed with pyelonephritis during pregnancy were abstracted for the dates of admission. The climatic records of the Tampa Bay area of Florida were obtained from the National Weather Service.

***Results:*** The average, minimum, or maximum daily temperature or average daily dew point during the month of admission had no significant effect on the rate of pyelonephritis during pregnancy in the Tampa Bay area.

***Conclusions:*** The rate of pyelonephritis during pregnancy per number of deliveries in the Tampa Bay area was not affected by the average, minimum, or maximum daily temperature or average daily dew point. © 1996 Wiley-Liss, Inc.

KEY WORDS

Weather, humidity, dew point, meteorology, pyelonephritis

Numerous reports have appeared in the literature linking disease states and meteorologic factors such as weather, humidity, and heat. Seasonal variations have been reported in regard to pregnancy-related conditions including conception,[1] birth,[2–4] preeclampsia,[5,6] eclampsia,[7,8] ectopic pregnancy,[9] stillbirth,[10] preterm delivery,[11] and multiple gestations.[12,13] Seasonal changes also have been reported to occur in the composition of urine,[14,15] renal-stone formation,[16–18] levels of circulating T cells,[19] interferon production,[20] and symptomatic urinary-tract infections in women.[21] Seasonal variations also have been reported with sexually transmitted diseases including chlamydia[22] and gonorrhea.[23]

The purpose of this study was to determine if the seasonal variation in ambient temperature and the dew point in the Tampa Bay area of Florida affected the incidence of pyelonephritis during pregnancy.

### MATERIALS AND METHODS

The records from Tampa General Hospital were reviewed for the diagnosis of pregnancy with pyelonephritis during a 4-year period, 1989–1992. The date of admission was abstracted and the diagnosis of pyelonephritis confirmed. Pyelonephritis was diagnosed if the patient had bacterial growth on a urine culture and signs and symptoms of pyelonephritis. The latter included chills, abdominal pain, backache, nausea, dysuria, fever, and costovertebral-angle tenderness on examination.

The climatic records of the Tampa Bay area of Florida were obtained from the National Weather

Address correspondence/reprint requests to Dr. John D. Busowski, Department of Obstetrics and Gynecology, University of South Alabama, 2451 Fillingim Street, Mobile, AL 36617.

Received November 22, 1995
Accepted February 14, 1996

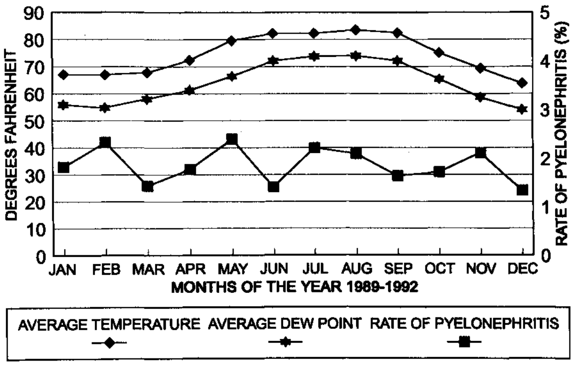

Fig. 1. Average monthly mean temperature and dew point compared with monthly rate of pyelonephritis per number of deliveries during the 4-year period 1989–1992.

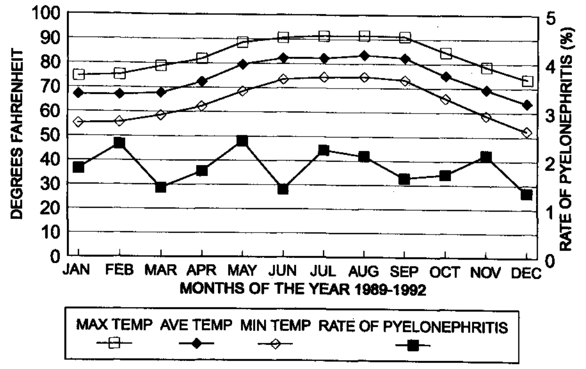

Fig. 2. Average monthly mean, minimum, and maximum temperatures compared with monthly rate of pyelonephritis per number of deliveries during the 4-year period 1989–1992.

Service. The mean, minimum, and maximum daily temperatures and the mean daily dew point were abstracted. The monthly and the 4-season averages were calculated. The data were analyzed using analysis of variance (ANOVA), the Komolgorov-Smirnov goodness of fit test, and Spearman's rank correlation test. $P < 0.05$ was considered statistically significant.

### RESULTS

There were 26,880 deliveries during the 48 months studied. There were 499 cases of pyelonephritis during pregnancy, for an incidence of 1.8%. The average temperature range was 65.6°F (18.67°C) to 82.9°F (28.28°C), with a mean yearly temperature of 74.1°F (23.39°C). The minimum monthly temperature ranged from 52.4°F (11.34°C) to 74.5°F (23.62°C). The maximum monthly temperature ranged from 73.6°F (23.12°C) to 91.6°F (33.12°C). The range of dew points was 54.1°F (12.28°C) to 74.0°F (23.34°C), with a mean average dew point of 63.9°F (17.73°C). No statistically significant correlation was found in the average, minimum, or maximum daily temperature or average daily dew point during the month of admission and the rate of pyelonephritis per number of deliveries (Figs. 1, 2).

### DISCUSSION

The observed incidence of pyelonephritis during pregnancy in our study was 1.8%, which is consistent with the range of 1–2.5% reported in the literature.[24–26] We anticipated that high ambient temperatures and dew points would affect the incidence of pyelonephritis in pregnancy in our community. Both are associated with increased perspiration and insensible water loss. If oral fluid intake is not adequate, relative dehydration occurs, with a resultant decreased urine production, less frequent voiding, increased urinary osmolarity, and increased solute production. The urinary pH and excretion of uric acid, potassium, magnesium, and sodium are reduced during the summer months.[14,15] The urinary pH and solute excretion affect the ability of the urine to remain sterile.[27] Decreased urinary pH and decreased solute excretion alone with less frequent voiding would increase the risk of lower urinary-tract infection, hence, the risk for pyelonephritis.

Our data do not support our initial hypothesis. We can postulate that this same analysis would detect a relationship between weather and pyelonephritis in a geographic area with greater variations in monthly temperatures and dew points, as was reported to occur for symptomatic bacterial urinary infections in Canada.[21] However, we question if it is possible to identify the effects of climatic factors on physiologic and pathologic processes in our current society. For many individuals, the increased prevalence of temperature- and humidity-controlled environments in the home, vehicles of transportation, and workplace minimizes prolonged exposure to external elements. Therefore, the ability of an investigator to isolate the impact of changes in weather conditions on a clinical phenomenon may be limited, if not unattainable.

### REFERENCES

1. Becker S: Seasonal patterns of births and conception. In Zorgniotti AW (ed): Temperature and Environmental Effects on the Testis. New York: Plenum Press, pp 59–72, 1991.
2. Guptill K, Berendes H, Forman MR, et al.: Seasonality of births among Bedouin Arabs residing in the Negev Desert of Israel. J Biosoc Sci 22:213–223, 1990.
3. Lam DA, Miron JA: Temperature and the seasonality of births. In Zorgniotti AW (ed): Temperature and Environmental Effects on the Testis. New York: Plenum Press, pp 73–88, 1991.
4. Russel D, Douglas AS, Allan TM: Changing seasonality of birth—A possible environmental effect. J Epidemiol Community Health 47:362–367, 1993.
5. Magann EF, Perry KG, Morrison JC, Martin JN: Climatic factors and preeclampsia-related hypertension disorders of pregnancy. Am J Obstet Gynecol 172:204–205, 1995.
6. Tan GWT, Salmon YM: Meterological factors and preeclampsia. Singapore Med J 29:133–137, 1988.
7. Neela J, Raman L: Seasonal trends in the occurrence of eclampsia. Natl J India 6:17–18, 1993.
8. Dieckmann WJ: The geographic distribution and effect of climate on eclampsia, toxemia of pregnancy, hyperemesis gravidarum, and abruptio placentae. Am J Obstet Gynecol 36:623–631, 1938.
9. Goldenberg M, Bider D, Seidman DS, Seidman DS, Lipitz S, Mashiach S, Oelsner G: Seasonal patterns in tubal pregnancy. Gynecol Obstet Invest 35:149–151, 1993.
10. Torrey EF, Bowler AE, Rawlings R, Terrazas A: Seasonality of schizophrenia and stillbirths. Schizophr Bull 19:557–562, 1993.
11. Cooperstock M, Wolfe RA: Seasonality of preterm birth in the collaborative perinatal project: Demographic factors. Am J Epidemiol 124:234–241, 1986.
12. Kamimura K: Epidemiology of twin births from a climatic point of view. Br J Prevent Soc Med 30:175–179, 1976.
13. Nonaka K, Miura T, Peter K: Low twinning rate and seasonal effects on twinning in a fertile population, the Hutterites. Int J Biometeorol 37:145–150, 1993.
14. Robertson W, Peacock M, Marshall R, Speed R, Nordin BEC: Seasonal variations in the composition of urine in relation to calcium stone-formation. Clin Sci Mol Med 49:597–602, 1975.
15. Robertson WG, Hodgkinson A, Marshall DH: Seasonal variation in the composition of urine from normal subjects: A longitudinal study. Clin Chim Acta 80:347–353, 1977.
16. Baker PW, Coyle P, Bias R, Rofe AM: Influence of season, age, and sex on renal stone formation in South Australia. Med J Aust 159:390–392, 1993.
17. Curtin J, Sampson M: Greenhouse effect and renal calculi. Lancet 23:1110, 1989.
18. Bateson EM: Renal tract calculi and climate. Med J Aust 2:111–113, 1973.
19. Levi FA, Canton C, Touitou Y, Reinberg A, Mathe G: Seasonal modulation of the circadian time structure of circulating T and natural killer lymphocyte subsets from healthy subjects. J Clin Invest 81:407–413, 1988.
20. Katila H, Cantell K, Appelberg B, Rimon R: Is there a seasonal variation in the interferon producing capacity of healthy subjects? J Interferon Res 13:233–234, 1993.
21. Anderson JE: Seasonality of symptomatic bacterial urinary infections in women. J Epidemiol Community Health 37:286–290, 1983.
22. Herold AH, Woodard LJ, Roetzheim RG, Pamies RJ, Young DL, Micceri T: Seasonality of *Chlamydia trachomatis* genital infections in university women. J Am Coll Health 42:117–120, 1993.
23. Ross JDC, Scott GR: Seasonal variation in gonorrhoea. Eur J Epidemiol 8:252–255, 1992.
24. Harris R: Acute urinary tract infections and subsequent problems. Clin Obstet Gynecol 27:874–890. 1984.
25. Wait R: Urinary tract infection during pregnancy. Postgrad Med 75:153–157, 1984.
26. Sweet R: Bacteriuria and pyelonephritis during pregnancy. Semin Perinatol 1:25–39, 1977.
27. Kaye D: Antibacterial activity of human urine: J Clin Invest 47:2374–2390, 1968.
